# Cryptic transmission of ST405 *Escherichia coli* carrying *bla*_NDM-4_ in hospital

**DOI:** 10.1038/s41598-017-18910-w

**Published:** 2018-01-10

**Authors:** Xiaoxia Zhang, Yu Feng, Weilong Zhou, Alan McNally, Zhiyong Zong

**Affiliations:** 1 Center of Infectious Diseases, West China Hospital, Sichuan University, Chengdu, China; 2 Division of Infectious Diseases, State Key Laboratory of Biotherapy, Chengdu, China; 3 Department of Infection Control, West China Hospital, Sichuan University, Chengdu, China; 4 Center for Pathogen Research, West China Hospital, Sichuan University, Chengdu, China; 5 Institute of Microbiology and Infection, College of Medical and Dental Science, University of Birmingham, Birmingham, UK

## Abstract

Three carbapenem-resistant *Escherichia coli* were recovered from rectal swabs of different patients in a tertiary hospital and were found carrying *bla*_NDM-4,_ an uncommon *bla*_NDM_ variant. Genome sequences of the isolates were obtained using Illumina technology and the long-read MinION sequencer. The isolates belonged to ST405 and phylogenetic group D, a globally distributed lineage associated with antimicrobial resistance. In addition to *bla*_NDM-4_, the three isolates carried 14 known resistance genes including the extended-spectrum β-lactamase gene *bla*_CTX-M-15_. There were only 1 or 2 SNPs between the isolates, suggesting a common origin and cryptic transmission in hospital. *bla*_NDM-4_ was located on a 46.5-kb IncFIA self-transmissible plasmid, which may facilitate further dissemination of *bla*_NDM-4_. Two copies of IS*26* bracketed a 14.6-kb region containing *bla*_NDM-4_ and have the potential to form a composite transposon for mediating the mobilization of *bla*_NDM-4_.

## Introduction

Carbapenem-resistant *Enterobacteriaceae* (CRE) have emerged as a major challenge to global public health. The production of carbapenem-hydrolyzing enzymes (carbapenemases) is the major mechanism mediating resistance to carbapenems in the *Enterobacteriaceae*. In *Escherichia coli*, NDM is the most common type of carbapenemase and has a few variants. NDM-4 has an amino acid substation (Met154Leu) compared with NDM-1, which leads to increased activity against carbapenems^1^. In China, *bla*_NDM-1_ and *bla*_NDM-5_ are the two most common types of *bla*_NDM_ variants in the *Enterobacteriaceae* ^2^, while *bla*_NDM-4_ remains uncommon. During an investigation on the prevalence of carbapenemase genes in carbapenem-resistant *Enterobacteriaceae* in our hospital, we found a cluster of three *E. coli* clinical isolates carrying *bla*_NDM-4_, which are reported here.

## Methods and Materials

### Isolates and *in vitro* susceptibility

The three *E. coli* isolates were recovered from the rectal swabs of three different patients in 2015 (Table 1). The initial species identification and *in vitro* antimicrobial susceptibility tests were performed by Vitek II (bioMérieux, Marcy-l'Étoile, France). In addition, MICs of amikacin, aztreonam, ceftazidime, ciprofloxacin, colistin, imipenem, meropenem, piperacillin-tazobactam, tigecycline and trimethoprim-sulfamethoxazole against the isolates were determined using the broth dilution method of the Clinical Laboratory Standards Institute (CLSI)^3^.

**Table 1:** Patient demographic data and diseases.

Patient	Sex	Age	Isolate	Days between ICU admission and *bla*_NDM-4_ positive swab collection	Length of hospital stay, days (date)	Diseases	Ward
1	Male	70	WCHEC96200	0	70 (8.19–10.27)	Diarrhea of unknown origin	General ICU
2	Male	57	WCHEC1837	3	22 (8.17–9.07)	Primary peritonitis, pneumonia	General ICU
3	Female	51	WCHEC99540	10	27 (8.25–9.20)	Liver cancer	Surgical ICU

### Carbapenemase gene screening and phylogenetic group typing

Acquired carbapenemase-encoding genes *bla*_GES_, *bla*_KPC_, *bla*_IMP_, *bla*_NDM_, *bla*_OXA-48_ and *bla*_VIM_ were screened using PCR as described previously^4–7^. The phylogenetic group for the isolates were determined using PCR as described previously^8^.

### Mating

Filter-based conjugation experiments were performed using the azide-resistant *E. coli* strain J53 as the recipient and 2 μg/ml meropenem plus 150 μg/ml sodium azide for selecting transconjugants. The presence of *bla*_NDM-4_ in transconjugants was confirmed by PCR.

### Pulse-field gel electrophoresis (PFGE)

The three isolates were subjected to PFGE following the protocol developed by the Centers for Disease Control and Prevention (Atlanta, GA, USA)^9^ but with different electrophoresis conditions. Whole-cell DNA from overnight cultures was embedded in 1% InCert agarose plugs, which were digested with 1 mg/L proteinase K and were then restricted with *Xba*I. PFGE electrophoresis was performed with 1% (w/v) PFGE grade agarose using a CHEF DRII system (Bio-Rad, Hercules, CA, USA) with a 6-V/cm current of 12 h at switch time of 5 to 40 s followed by 8 h at switch time of 3 to 8 s^10^.

### Genome sequencing and analysis

Genomic DNA of the three isolates was prepared using the QIAamp DNA Mini Kit (Qiagen, Hilden, Germany) and was subjected to whole genome sequencing with 150 × coverage using the HiSeq X10 Sequencer (Illumina, San Diego, CA). Reads were trimmed using Trimmomatic^11^ and were then assembled to contigs using the SPAdes program^12^ with careful mode turned on. Sequence types were determined using the genomic sequence to query the multi-locus sequence typing database of *E. coli* (http://enterobase.warwick.ac.uk/species/index/ecoli). Antimicrobial resistance genes were identified from genome sequences using the ABRicate (https://github.com/tseemann/abricate) program. Plasmid replicon types were determined using by the PlasmidFinder tool at http://genomicepidemiology.org/ and the allele types of IncF plasmids were assigned using the IncF replicon typing tool^13^.

To determine the clonal relatedness of the three isolates, the three genomes were aligned using the Harvest Suite^14^ with default settings. Single nucleotide polymorphisms (SNPs) on recombination sites were removed by the Gubbins program^15^.

To facilitate circulating the plasmid sequence, strain WCHEC96200 was also sequenced using the long-read MinION Sequencer (Nanopore, Oxford, UK), which generated 477.161 reads (30.9 GB) and was converted into a single fastq file of 2 GB using poretools^16^. The assembly of reads were performed using Canu^17^ with default settings. Circlator^18^ was then used to locate and circularize complete chromosome and plasmids in the draft assembly. Contigs representing the chromosome and plasmids were subsequently polished using Nanopolish (https://github.com/jts/nanopolish) combined with BWA-MEM^19^. The polished genome of strain WCH96200 was cured by quality-trimmed Illumina reads using Pilon^20^ with default settings, to eventually obtain a more accurate assembly.

Nucleotide sequence accession numbers. Draft whole-genome sequences of isolates WCHEC1837, WCHEC96200 and WCHEC99540 have been deposited into GenBank under the accession numbers NGUU00000000, NGUV00000000 and NGUW00000000, respectively. The complete sequences of pNDM4_WCHEC96200 has been deposited into GenBank under the accession number CP022226.

## Results and Discussion

The three isolates were all resistant to ampicillin-sulbactam, aztreonam, cefepime, ceftazidime (MIC, >256 μg/ml), ciprofloxacin (MIC, >256 μg/ml), ertapenem, gentamicin, imipenem (MIC, 64 μg/ml), levofloxacin, meropenem (MIC, 64 μg/ml), nitrofurantoin, piperacillin-tazobactam, tobramycin and trimethoprim-sulfamethoxazole but were susceptible to amikacin (MIC, 8 μg/ml for isolate from the first patient or 16 μg/ml for isolates from the other two patients), colistin (MIC, 1 μg/ml) and tigecycline (MIC, < 0.25 μg/ml).

The three isolates had *bla*_NDM_ only, which was identified as *bla*_NDM-4_ by amplifying and sequencing the complete coding sequence of *bla*_NDM_ using additional primers^4^. In addition to *bla*_NDM-4_, the three isolates had the same 14 intact antimicrobial resistance genes mediating resistance to aminoglycosides (*aac(6′)-Ib-*cr, *aac(3)-IIa*, *aadA5*, *strA* and *strB*), β-lactams (*bla*_CTX-M-15_ and *bla*_OXA-1_), macrolides (*mph(A)*), phenicol (*floR*), quinolones (*aac(6′)-Ib-*cr), tetracycline (*tet(A)* and *tet(B)*), sulphonamides (*sul1* and *sul2*) and trimethoprim (*dfrA17*) in their whole genome sequences (see below).

A total of 4,670,485 to 5,014,495 reads were generated for the three isolates, which were then assembled to 170 to 174 contigs (144 to 147 were ≥1,000 bp in length) with a 50.61 to 50.64% GC content, respectively.

The three isolates belonged to ST405 and phylogenetic group D. ST405 *E. coli* has a global distribution and is typically associated with extended-spectrum β-lactamases (ESBLs) such as CTX-M-15^21^, as seen in the three isolates here. Although *bla*_NDM-4_ remains uncommon, its association with ST405 *E. coli* has been previously documented. Six ST405 *E. coli* carrying *bla*_NDM-4_ found in Italy were introduced from India^22^ and an ST405 *E. coli* carrying *bla*_NDM-4_ was found in a Danish patient who had been previously hospitalized in Vietnam^23^. Unfortunately, their genome sequences are not available for comparison.

The three isolates had identical PFGE patterns (data not shown). Indeed, there were only 1 or 2 SNPs between the isolates, suggesting very recent acquisition from a common source or recent direct transmission. To investigate this further, the three patients were ordered according to the date on which they provided a positive swab for *bla*_NDM-4_-carrying *E. coli*. All of the three patients were admitted to our hospital in August 2015. The first and second patients were hospitalized in a 50-bed general ICU, while the third was hospitalized in a 30-bed surgical ICU. The hospital stay periods of the three patients were overlapped (Table 1). The first patient was transferred from another local hospital and *bla*_NDM-4_-carrying *E. coli* was detected from the first patient on the same day of his admission to our hospital, suggesting that the isolate was very likely introduced from another hospital. The rectal swab of the second patient that was collected on admission to ICU did not grow *bla*_NDM-4_-carrying *E. coli*, while that collected on the third day of her ICU stay did, suggesting that the *bla*_NDM-4_-carrying *E. coli* was acquired in the ICU. The third patient had not stayed in the local hospital from which the first and second patients were transferred and was admitted to the Liver Surgery Ward in our hospital. Unfortunately, no rectal swabs were collected during his 10-day stay in the surgery ward. The rectal swab collected on admission to the surgical ICU grew *bla*_NDM-4_-carrying *E. coli*. It is possible that the isolate was carried by the patient on admission to our hospital or was acquired during his stay in the Liver Surgery Ward. Nonetheless, the very few SNPs between the isolate of the third patient and those of the other patients suggest that the isolate of the third patient was acquired within our hospital, though the exact route of the acquisition is not clear. No evident epidemiological links could be identified between the third patient and the other two patients. However, as the three patients had overlapped stay in our hospital, there were many possible yet-to-be-identified indirect contacts between the three patients including movement of staff, equipment, or seeding of the wider hospital environment by the strain.

**Figure 1:**
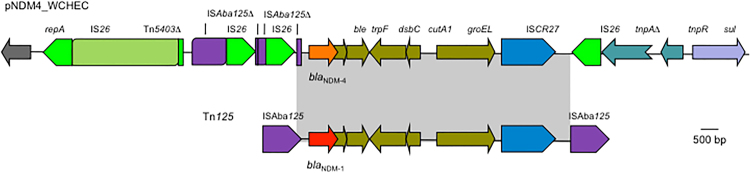
The genetic context of *bla*_NDM-4_ on pNDM4_WCHEC96200. The IS*Aba*125-formed composite transposon Tn*125* carrying *bla*_NDM-1_ is shown for comparison with identical regions being indicated by grey. Δ refers to truncated genes or elements. *tnpA*Δ and *tnpR* belong to a transposon of the Tn3 family and *sul* encodes a sodium-independent anion transporter. *repA* encodes the replication initiation protein of the IncFIA replicon.

In the three isolates, *bla*_NDM-4_ was carried by self-transmissible plasmids. Transconjugants carrying *bla*_NDM-4_ were resistant to imipenem and meropenem (MICs, 16 μg/ml). The complete sequence of the plasmid carrying *bla*_NDM-4_, designated pNDM4_WCHEC, here in strain WCHEC96200 was therefore also obtained. Sequences of *bla*_NDM-4_-carrying plasmids in the other two isolates were circularized by PCR mapping using pNDM4_WCHEC96200 as the template and were actually identical to that of pNDM4_WCHEC96200. pNDM4_WCHEC96200 is 46.5-kb and contains an IncFIA replicon, which belongs to the IncFIA allele 13, and an additional gene encoding a replication initiation protein of the RepB family, which was not assigned an replicon type by the PlasmidFinder. *bla*_NDM-4_ was located in a 14.6-kb region bracketed by two copies of IS*26* (Fig. 1). In this region, there are Tn*5403*Δ (a truncated transposon of the Tn*3* family), two IS*Aba125*, both of which were interrupted by the insertion of IS*26*, *bla*_NDM-4_ *, ble* (mediating bleomycin resistance), *trpF* (encoding the phosphoribosylanthranilate isomerase), *dsbC* (encoding a *tat* twin-arginine translocation pathway signal sequence domain protein), *cutA1* (encoding a periplasmic divalent cation tolerance protein), *groES*/*groEL* (encoding a chaperonin) and IS*CR27* (Fig. 1). The two copies of IS*26* have the potential to form a composite transposon, which could mobilize the intervening genetic components including *bla*_NDM-4_ in this case. The genetic context of *bla*_NDM-4_ is highly similar to that of *bla*_NDM-1_. It is common that *bla*_NDM-1_, *ble*, *trpF*, *dsbC*, *cutA1*, *groES*/*groEL* and IS*CR27* are bracketed by two copies of IS*Aba*125, which form a composite transposon termed Tn*125* (Fig. 1). It is therefore likely that *bla*_NDM-4_ has evolved from *bla*_NDM-1_ in such a genetic context.

*bla*_NDM-4_ has been found on plasmids of IncF^22,24^, IncK^25^, IncL/M^26^ or IncX3^27,28^. However, the complete sequence of IncF plasmids carrying *bla*_NDM-4_ is not available for comparison. Nonetheless, pNDM4_WCHEC96200 appeared to be a new plasmid that is most closely related (58% coverage and up to 99% identity) to the plasmid tig00001145_pilon (GenBank accession no. CP021881), which contains two replicons, IncFII and IncR, of *E. coli* AR_0137. pNDM4_WCHEC96200 contains components with significant similarity with several plasmids of different replicon types, suggesting a mosaic composition. A 8.1-kb region containing a gene encoding ATPase is 99% identical to the corresponding region of several plasmids including an IncR plasmid pKPN-041 (GenBank accession no. CP014758). A 7.5-kb region containing a gene encoding sulfate transporter is 99% identical to an IncFII and IncFIA plasmid pCAV1043-51 (GenBank accession no. CP011587).

In conclusion, we identified the in-hospital transmission of an ST405 *E. coli* strain carrying *bla*_NDM-4_, an uncommon variant of *bla*_NDM_. The association of *bla*_NDM-4_ with a globally distributed clone, ST405 in this case, is worrisome. The self-transmissible IncFIA plasmid carrying *bla*_NDM-4_ is a new mosaic plasmid, which could mediate the dissemination of *bla*_NDM-4_ into other *E. coli* strains or even other species of the *Enterobacteriaceae*. The *bla*_NDM-4_ gene was bracketed by two copies of IS*26*, which have the potential to mobilize *bla*_NDM-4_ by hijacking more plasmids as the vehicle to disseminate this gene.
